# Molecular mechanisms of syncytin-1 in tumors and placental development related diseases

**DOI:** 10.1007/s12672-023-00702-6

**Published:** 2023-06-16

**Authors:** Qianqian Wang, Ying Shi, Qiang Bian, Naibin Zhang, Meng Wang, Jianing Wang, Xuan Li, Luhao Lai, Zhankui Zhao, Honglian Yu

**Affiliations:** 1 Department of Biochemistry, Jining Medical University, 133 Hehua Road, Jining, 272067 Shandong People’s Republic of China; 2 Collaborative Innovation Center, Jining Medical University, Jining, 272067 Shandong People’s Republic of China; 3 Department of Pathophysiology, Weifang Medical University, Weifang, 261053 Shandong People’s Republic of China; 4 The Affiliated Hospital of Jining Medical University, Jining Medical University, 89 Guhuai Road, Jining, 272029 Shandong People’s Republic of China

**Keywords:** Syncytin-1, Biomarker, Molecular mechanism, Signal pathway, Placenta

## Abstract

Human endogenous retroviruses (HERVs) have evolved from exogenous retroviruses and account for approximately 8% of the human genome. A growing number of findings suggest that the abnormal expression of HERV genes is associated with schizophrenia, multiple sclerosis, endometriosis, breast cancer, bladder cancer and other diseases. HERV-W env (syncytin-1) is a membrane glycoprotein which plays an important role in placental development. It includes embryo implantation, fusion of syncytiotrophoblasts and of fertilized eggs, and immune response. The abnormal expression of syncytin-1 is related to placental development-related diseases such as preeclampsia, infertility, and intrauterine growth restriction, as well as tumors such as neuroblastoma, endometrial cancer, and endometriosis. This review mainly focused on the molecular interactions of syncytin-1 in placental development-related diseases and tumors, to explore whether syncytin-1 can be an emerging biological marker and potential therapeutic target.

## Introduction

Human endogenous retroviruses were acquired through multiple infections and integration of extinct exogenous retroviruses during primate evolution. This ancestral infection specifically affects the germline, and the resulting endogenous retroviral proviruses can transmit vertically. HERVs become a stable part of the human genome by continuous Mendelian inheritance, accounting for approximately 8% of human DNA [1, 2]. According to the types of tRNA recognized by the primer-binding site [3], HERV can be divided into at least 31 families, including HERV-W, HERV-T [4], HERV-K [5], HERV-F [6], HERV-E [7] and so on. Although HERV family members have not been identified as replication-competent, they share substantial structure similarity with exogenous retroviruses, for example, HERV-K retains three of the four intact ORFs with the potential to encode proteins or peptides [8]. Most genes in HERV families are silenced, but some genes remain their function. Studies have found abnormal expression of HERV is associated with various diseases such as prostate cancer [9], breast cancer [10], AIDS [11], colorectal cancer [12], multiple sclerosis [13] and neurodegenerative disorders [14].

**Fig. 1:**
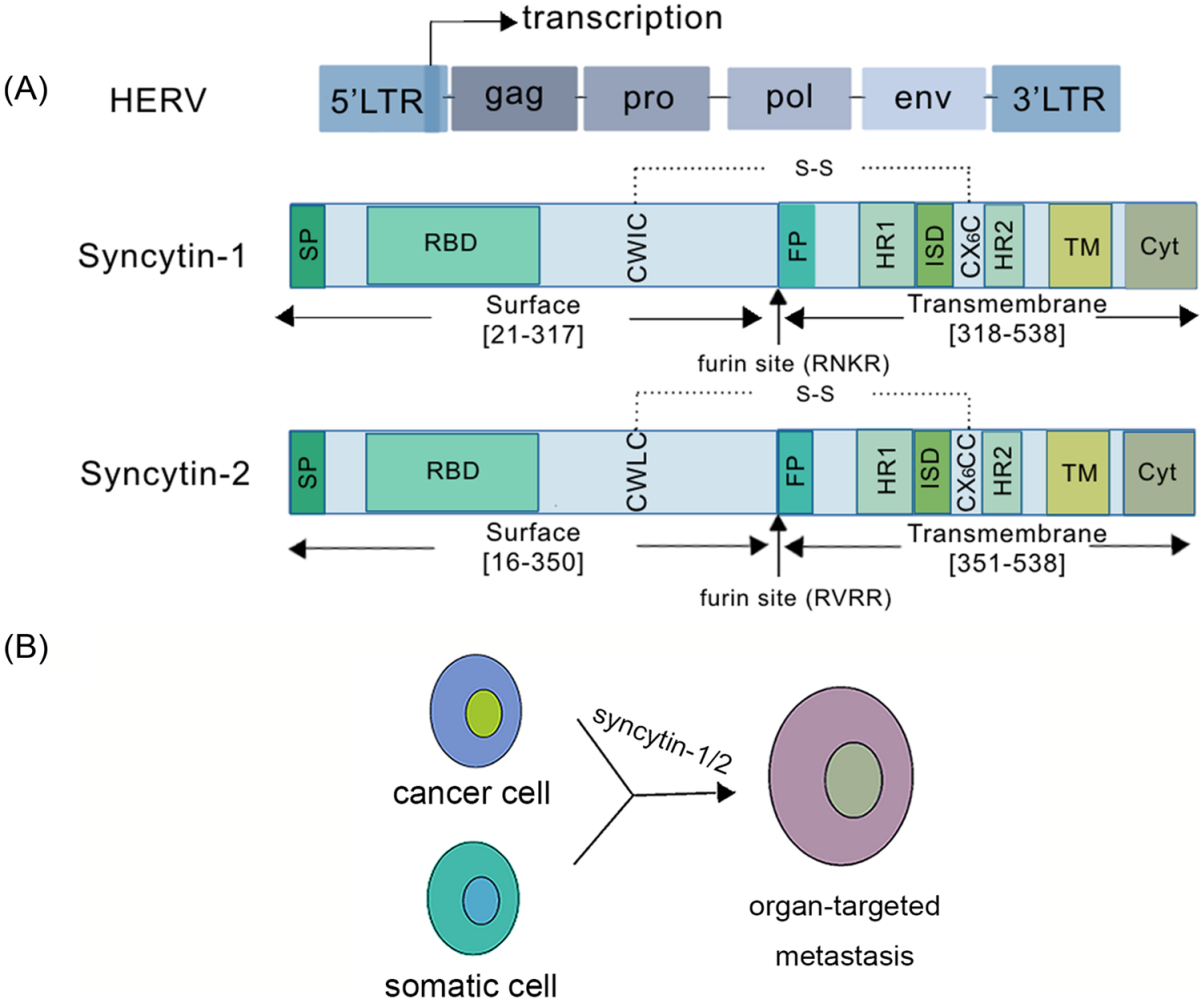
Structure and function of syncytin-1. **A** The general structure of HERV consists mainly of two identical LTR sequences and four typical viral genes, gag, pro, pol and env. And there is a transcriptional promoter at the end of the 5'LTR. The structure of syncytin-1 and syncytin-2 mainly includes SU domains and TM domains. **B** Syncytin-1 could promote the fusion of somatic cells and cancer cells

The classical HERV provirus structure consists of two identical long terminal repeats (LTRs) and four typical viral genes, gag, pro, pol and env (Fig. 1A) [15]. LTRs have the promoter and transcription termination signals to regulate the expression of the virus gene and adjacent gene [16–21]. The gag gene encodes a viral core structural protein [22]. It can participate in viral RNA encapsidation and particle formation [23]. The abnormal expression of gag may be associated with pituitary adenomas [24] and lichen planus [25]. The pol gene encodes viral enzymes such as reverse transcriptase and integrase [26–28], which abnormal expression may be related to mixed connective tissue disease and systemic sclerosis [29]. The env gene encodes viral envelope glycoprotein, which is important for receptor recognition and membrane fusion [28]. Its abnormal expression is greatly associated with lymphoma [30], melanoma [31], non-small cell lung cancer [32], schizophrenia [33] and leukemia [34].

Env is a membrane glycoprotein and forms the spike glycoprotein on the surface of retroviral particles [35]. Syncytin-1 is an env protein of the HERV-W family, which receptor is solute carrier family 1 Member 4 and 5 (SLC1A4 and SLC1A5) [36], and syncytin-2 is an env protein of the HERV-FRD family, which receptor is major facilitator superfamily domain containing 2A (MFSD2A) [37]. They are homologous membrane glycoproteins with a similar structure, involved in forming the placental syncytiotrophoblast (Fig. 1A) [38]. Syncytin-1/2 could promote cell fusion effect between tumor cells and normal cells, and might be associated with tumor metastasis (Fig. 1B). Therefore, both syncytin-1 and syncytin-2 can play important roles in pathological and physiological processes.

According to published articles, no systematic analysis of the molecular mechanisms of syncytin-1 in placental development-related diseases and tumors has been reported. Therefore, we will focus on the networks and signal pathways of syncytin-1 during the placental development related diseases and tumors.

## The structure and function of syncytin-1

Syncytin-1 is a highly fusogenic membrane glycoprotein of the HERV-W, specifically expressed in the placental trophectoderm [39, 40]. It is mainly localized at locus 7q21.2 [41], and also located in the acrosomal region and equatorial segment of the sperm [42]. The endoprotease furin can cleave the syncytin-1 polypeptide into surface (SU) domains and transmembrane (TM) domains at the RNKR site, which are linked by the intersubunit disulfide bond (S–S) [38]. A signal peptide (SP) and a receptor-binding domain (RBD) are in the amino-terminal protein of the SU domain. And the SU domain is responsible for receptor recognition and binding. The TM domain contains an immunosuppressive domain (ISD), which has immunosuppressive properties. This property could be involved in materno-fetal tolerance toward fetal tissue with the father's gene to survive in the mother's body [43]. The carboxyl-terminal R peptide of the TM domain, which cleaves during virion maturation, can activate the fusion function of the TM domain (Fig. 1A) [15, 29, 40, 44, 45]. Moreover, syncytin-1 can interact with SLC1A4 and SLC1A5, and mediate the formation of syncytium [46], which is the channel of fetal-maternal exchanges [47]. And syncytin-1 can induce infected cells to resist reinfection when interacting with the SLC1A5 receptor on cells. This phenomenon is known as superinfection interference [48]. In summary, the structure of syncytin-1 determines its fusion and immune properties.

## The regulation mechanisms of syncytin-1 in physiological processes

Studies found that syncytin-1 and its receptor SLC1A5 are localized at the acrosomal region and equatorial segment of the sperm, and SLC1A5 is also expressed in human oocytes [42]. Therefore, the interaction of syncytin-1 and its receptor could promote membrane fusion of sperm and egg cells, facilitating the formation of fertilized eggs. Syncytin-1 is also expressed in the trophectoderm underlying the inner cell mass in human trophoblast cells [49], indicating that syncytin-1 could play a vital role in embryo implantation. In addition, syncytin-1 shows dynamic expression during embryonic (fetal) development. The syncytin-1 mRNA is significantly increased since the late first trimester (9–12 weeks of gestation) compared to the early first trimester (5–7 weeks of gestation), but then significantly decreased in the late third trimester (37–40 weeks of gestation) [50]. As we all know, the main functions of the placenta are transport, metabolism, protection and endocrine [51]. These changes during different periods of gestation reflect the relationship between fetal development and placental demand. In a word, it is the structure and function of syncytin-1 that determine its importance in placental development.

The role of syncytin-1 in placental development could be affected by various factors. The expression of syncytin-1 is influenced by methylation of CpG islands in the 5′LTR [52], and DNMTs is involved in DNA methylation [53]. Transient DNA hypomethylation in the first trimester of pregnancy can promote syncytin-1-mediated cell fusion and differentiation, while DNA hypermethylation at term reduces syncytin-1-mediated cell fusion [54]. In addition, changes in posttranslational modification of histones are also regulating the expression of syncytin-1. Interestingly, histone modification and DNA methylation often coexist and affect each other [55]. Acetylate glia cells missing a (GCM1) is essential for placental development [56, 57], and it can promote syncytin-1-mediated trophoblast fusion [58]. Its transcript and phosphorylation levels of the GCM1 gene were activated by the cyclic adenosine monophosphate(cAMP)-protein kinase A(PKA) signal pathway and CREB-binding protein (CBP) plays an auxiliary role in this process. In addition, the increased acetylation, decreased ubiquitination, and improved stability of GCM1 protein were regulated by CBP. The changes in GCM1 expression and structure could upregulate syncytin-1-mediated trophoblast fusion [59]. Moreover, human chorionic gonadotrophin (hCG) and corticotropin-releasing hormone (CRH) all can activate cAMP-PKA signal pathway, regulating the expression of syncytin-1 [60–62]. Syncytin-1 is involved in the proliferation of cytotrophoblasts through cell cycle [28], and also mediates the fusion of the villous cytotrophoblast into multinucleated syncytiotrophoblasts [36, 63, 64], the insufficient expression levels of syncytin-1 may lead to defects in the structure and function of the syncytium. In addition, studies have shown that syncytin-1 could also have non-fusogenic activities by participating in the proliferation, differentiation and apoptosis of the trophoblast [65]. In summary, the expression of syncytin-1 is regulated by methylation, cAMP-PKA-GCM1 signal pathway. Syncytin-1 maintains the homeostasis balance in syncytium through its non-fusion and fusion functions, and plays a vital role in the human placenta.

Studies have shown that syncytin-1 can be present in trophoblast/placenta-derived microvesicles and shed from the placenta into the maternal circulation, involved in microvesicles-mediated activation of immune cells and immune cell responses to lipopolysaccharide stimulation [66]. The syncytin-1 protein not only affects early innate immune response but also influences adaptive response, therefore, increasing the susceptibility to infection [67]. The leukocytic syncytin-1 expressed in all four leukocyte types, blasts, granulocytes, lymphocytes and monocytes [68], and syncytin-1 can also promote the activation of monocytes [69]. Syncytin-1 in astrocytes and microglia can trigger the activation of c-reactive protein through the toll-like receptor 3 (TLR3)-Interleukin-6 (IL-6) signal pathway, inducing inflammatory responses [33]. In conclusion, syncytin-1, in addition to its role in the placenta, could also be involved in the occurrence of infections and immune responses.

## Syncytin-1 and placental development-associated diseases

### Syncytin-1 and preeclampsia

#### Expression of syncytin-1 in preeclampsia

Preeclampsia (PE) can lead to systemic vascular damage in liver, kidney and brain failure as the main manifestation, and is mainly characterized by hypertension and proteinuria during pregnancy [70]. Both poor trophoblast [71] and vascular dysfunction [72] are important pathological features of PE. Studies suggest that suboptimal maternal cardiovascular performance can lead to placental malperfusion, causing the occurrence of PE [73]. And PE is the main cause of maternal and fetal morbidity and mortality worldwide, accounting for approximately 4.6% of all births [74] and causing about 42,000 maternal deaths annually [75]. At present, the main established treatment for PE is to terminate the pregnancy and preterm labor, so the identification of reliable biomarkers is of great significance for the early detection and prevention of PE.

The syncytium is a continuous layer of syncytiotrophoblasts whose formation is mediated by syncytin-1 and has an important role in maternal–fetal circulation. Zhuang et al. performed RT-PCR analysis on placental tissues from 31 to 41 weeks, including 12 normal placental tissues and 8 PE placental tissues, and found syncytin-1 hypermethylation and decreased levels of gene expression in PE human placenta compared to normal human placenta [76]. Lee et al. performed in-situ hybridization and immunohistochemical analysis of 11 cases of 9–21 weeks early gestation human placental tissues and a total of 17 normal placental tissues and 21 PE placental tissues after delivery. They found that the expression of the syncytin-1 protein is down-regulated and the protein localization is abnormal in PE. Syncytin-1 protein is located in the plasma membrane of syncytiotrophoblast basal cells in normal placental tissue, while that in PE is localized in the apical syncytiotrophoblast microchorion [77]. Vargas et al. set up two experimental groups (moderated PE, n = 9 and severe PE, n = 7) and a control group (normal placenta, n = 8). They found a fusion dysfunction of primary trophoblast cells in PE patients through cell fusion assay. And the mRNA and protein levels of syncytin-1 all decreased in PE compared to normal placenta tissues by RT-PCR and Western blot (WB) [78]. In human primary cytotrophoblast cells and BeWo trophoblast cell lines, Ruebner et al. showed that the expression of syncytin-1 in PE is high [79]. Conversely, Holder et al. found the transcript level of syncytin-1 is higher in PE placentas than that in normal term placentas (Table 1) [80]. The different expression of the syncytin-1 gene in these studies is mainly due to the following reasons. Firstly, an anti-syncytin-1 SU polyclonal antibody (H-280, rabbit, Santa Cruz) was used in the Holder et al. study [80], but two polyclonal anti-syncytin-1 antibodies (GeneTex, Orbigen) were used in Lee et al. study [77] and Vargas et al. study respectively [78]. Secondly, it may be due to the different periods in which the placenta samples were taken. First and second trimester placentas and term placentas were used in experiments with high expression of syncytin-1. Zhuang et al. proved that the expression of syncytin-1 is low with placentas in gestational age ranging from 31 to 41 weeks. In addition, racial differences should also be discussed. In conclusion, whether syncytin-1 can be used as a potential marker of PE needs further study.

**Table 1:** Relationship between expression of syncytin-1 and preeclampsia

Type of cancer	Specimen	Stage(w)/Number of cases	Area	Methods	Upstream factors	Expression	Ref.
Preeclampsia	Tissue	9-21w/11 late/(N:17,P:21)	USA Belgium	in-situ hybridization IHC	unk	Low	[77]
	Cell	unk/unk	Germany	RT-PCR sqPCR ELISA WB	PPARγ/RXRα	Low	[79]
	Tissue	N/(n = 8) MPE/(n = 9) SPE/(n = 7)	Canada	cell fusion assay RT-PCR WB	unk	Low	[78]
	Tissue	31-41w/(N:12,P:8)	USA	RT-PCR	DNMT1 DNMT3B3 Hypermethylation	Low	[76]
	Tissue	First trimester/unk second trimester/unk term/unk	UK	qRT-PCR WB	unk	Slightly high	[80]

*unk* unknown; *P* preeclampsia; *N* normal; *CT* cytotrophoblast; *WB* Western Blot; *MPE* moderated preeclampsia; *SPE* severe preeclampsia

#### The role and mechanism of syncytin-1 in preeclampsia

**Fig. 2:**
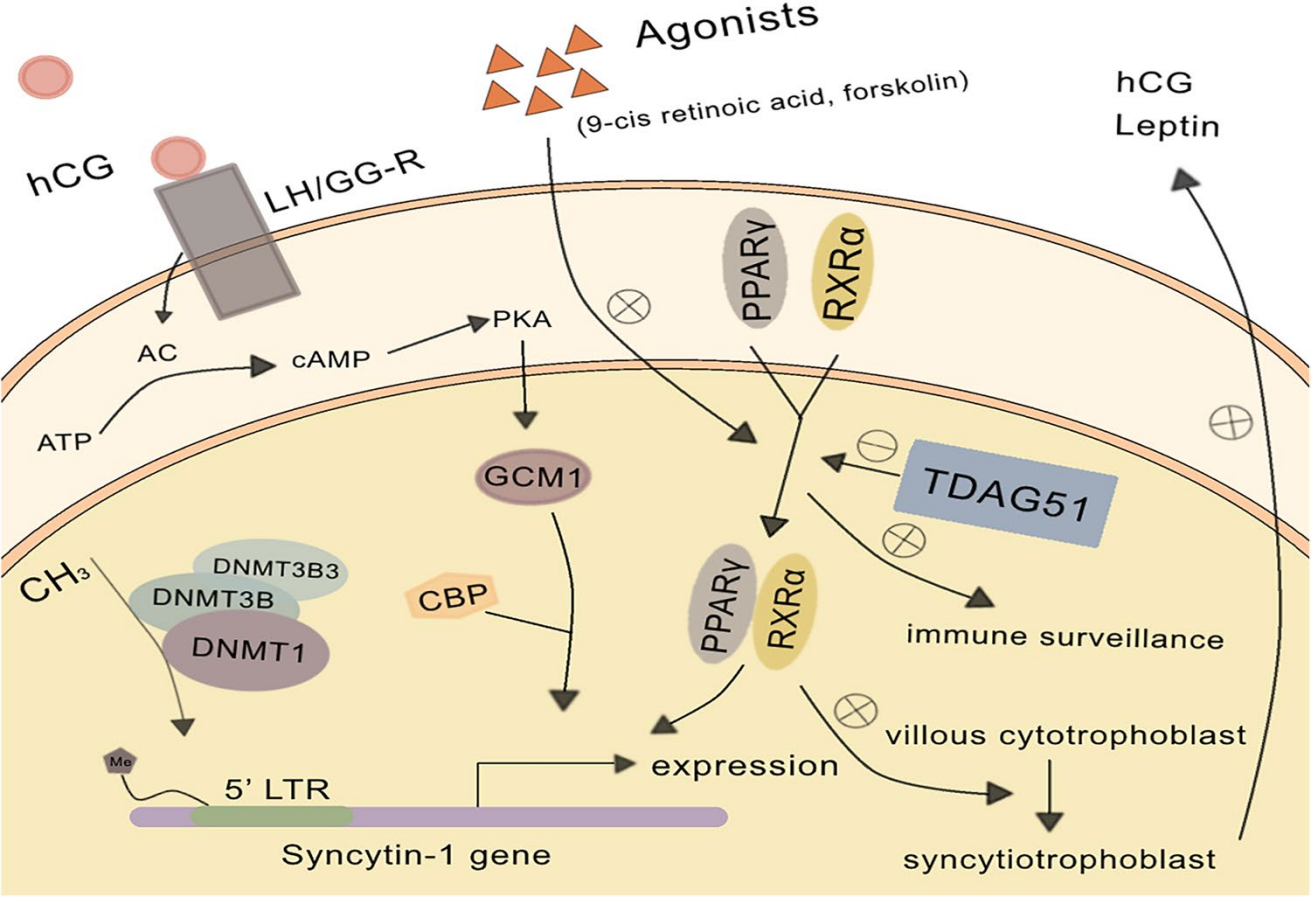
Molecular mechanisms and signal pathways of syncytin-1 expression in preeclampsia (PE) Induction of DNA methylation by the DNA methyltransferases DNMT1 and DNMT3B can reduce syncytin-1 expression. Human chorionic gonadotrophin (hCG) could regulate syncytin-1 expression by activating cAMP-PKA-GCM1 signal pathway. PPARγ and RXRα can form heterodimers, and their activation allows muscle-invasive bladder cancer to evade immune surveillance. PPARγ/RXRα nuclear hormone receptor activators such as 9-cis retinoic acid and forskolin can also reduce the expression of syncytin-1 and promote the fusion of villous cytotrophoblast into syncytiotrophoblast. T-cell death-associated gene 51 (TDAG51) is a potential inhibitor of PPARγ/RXRα

Syncytin-1 can play a role in PE through multiple pathways (Fig. 2). Research shows that the placental syncytin-1 protein in PE is located in the apical syncytiotrophoblast microchorion, while it is normally located in the plasma membrane of the syncytiotrophoblast [77]. These results suggest that abnormal protein localization is associated with the occurrence of PE.

Syncytin-1 is regulated by nuclear hormone receptor peroxisome proliferator-activated receptor γ (PPARγ)/retinoid X receptor α (RXRα). The nuclear receptor PPARγ and RXR can form heterodimers and localize in the promoter region to play multiple roles. T-cell death-associated gene 51 (TDAG51) is a negative regulator of PPARγ, competitively binds PPARγ with RXRα, blocks the formation of heterodimers, and thereby inhibits adipogenesis [81]. PPARγ/RXRα genome activation in muscle-invasive bladder cancer can evade immune surveillance [82]. Ruebner et al. found that PPARγ/RXRα activators such as troglitazone, forskolin, 9-cis retinoic acid could increase the expression of syncytin-1 in cytotrophoblast cells, while the inhibitor SB203580 decreased the expression of syncytin-1. The expression of syncytin-1 was significantly decreased in BeWo cell lines treated with activator troglitazone and inhibitor SB203580, which was significantly increased by treatment with inhibitors 9-cis retinoic acid and forskolin. PPARγ/RXRα signal gene expression analysis was performed on the placental tissues, and it was found that the mRNA level and protein expression of syncytin-1 in PE were lower than those in normal placental tissue. Therefore, PPARγ/RXRα signaling directly upregulates syncytin-1 expression and cell fusion in human primary cytotrophoblast and BeWo trophoblasts, promoting the formation of syncytiotrophoblast and the development of the placenta [79]. hCG, leptin, resistin and xenobiotic transporter ABCG2 are all specific PPARγ target genes for placental development. hCG could associate with the fusion of cytotrophoblast cells [83]. Leptin can be produced in syncytiotrophoblast and endothelial cells of the placenta [84, 85]. Maternal placental leptin protein and mRNA levels are significantly increased in PE [86]. ABCG2 is down-regulated in pregnancies with PE further complicated by HELLP (hemolysis, elevated liver enzymes and low platelets) syndrome [87]. Consequently, syncytin-1 could promote the fusion of villous cytotrophoblast into syncytiotrophoblast, and the synthesis of hCG and leptin through regulation by PPARγ/RXRα.

DNA methyltransferase 3 Beta 3 (DNMT3B3) is an inactive isoform, it can stimulate the activities of DNMT3B in vitro [88]. Studies have found that the methylation level of PE is higher than that in normal placental tissues. With the high expression of DNMT3B and DNA methyltransferase 1 (DNMT1), the 5′LTR region of syncytin-1 is hypermethylated, and the expression of syncytin-1 is decreased [76]. Studies have shown a correlation between low expression of syncytin-1 and high apoptosis of cells [89]. This could also be a cause of abnormal placental development in patients with PE.

Based on most experimental results, the expression of syncytin-1 is down-regulated in PE. PPARγ/RXRα, DNMT1 and DNMT3B3 are all factors to affect the expression of syncytin-1. But the controversial results still need to be studied.

### Syncytin-1 and other diseases related to placental development

The abnormal expression of syncytin-1 is also associated with other placental developmental related diseases in addition to PE. Studies have found that the expression of syncytin-1 and its receptor SLC1A5 have significantly decreased in samples with asthenozoospermic, oligozoospermic and oligoasthenozoospermic than normozoospermic samples [90], suggesting that decreased expression of syncytin-1 and SLC1A5 could be a cause of infertility. So, it might be one way to treat infertility in the future by increasing their expression. The mRNA and protein levels of syncytin-1 are lower in intrauterine growth restriction (IUGR) placentas than in normal placentas, inducing cell fusion abnormally and apoptosis increasingly [39, 79, 89, 91]. Syncytin-1 glycoprotein is significantly enhanced at the apical of the syncytiotrophoblast of hydatidiform moles compared with the normal placenta [92], indicating that the abnormal expression and localization of syncytin-1 lead to abnormal placental development. Therefore, syncytin-1 can potentially be a molecular marker for these diseases, but further investigation of the specific molecular mechanisms is needed.

## Syncytin-1 and tumors

### Syncytin-1 and neuroblastoma

#### Expression of syncytin-1 in neuroblastoma

Neuroblastoma (NB) is the most common extracranial solid tumor in infants and children, accounting for more than 15% of cancer-related deaths in children [93]. And it is caused by developing sympathetic NB cells with a high degree of malignancy and unique biological heterogeneity [94]. Radiation therapy is now the standard of care for high-risk NB, and this treatment is important for local lesion clearance and prevention of local recurrence [95], but the detection of other metastatic lesions in vivo and the degree of prognosis remain unclear. Treatment failure and poor prognosis are often marked by resistance to chemotherapy or immunotherapy in patients with advanced metastatic NB [96]. Therefore, the identification of reliable biomarkers is of great significance for understanding and treating NB.

All these studies have shown that syncytin-1 is highly expressed in NB cell lines (Table 2). Chen et al. and Li et al. all found that the expression of syncytin-1 in NB cell lines SH-SY5Y and IMR-32 was significantly higher than that in normal cells [97, 98]. Hu et al. came to the same conclusion in NB cell lines SH-SY5Y, IMR-32, SK-N-DZ [99], Wieland et al. in SH-SY5Y, IMR-32, SiMa [96], and Liu et al. in SH-SY5Y [100]. Although syncytin-1 is overexpressed in NB cell lines, further studies in NB tumor tissues are needed in the future.

**Table 2:** Relationship between high expression of syncytin-1 and neuroblastoma

Type of cancer	Specimen	Cell lines	Methods	Upstream factors	Downstream factors	Signal pathway	Oncogenic effect	Ref.
Neuroblastoma	Cell	SH-SY5Y	Semi-qPCR qRT-PCR WB	Caffeine aspirin	unk	unk	Proliferation	[100]
	Cell	SH-SY5Y SK-N-AS SK-N-DZ	qRT-PCR	Oxygen tension DNA methylation	unk	unk	Proliferation	[99]
	Cell	SH-SY5Y IMR-32 SiMa	qRT-PCR	Culture medium micro-environmnt	unk	unk	Proliferation invasion	[96]
	Cell	SH-SY5Y IMR-32	qPCR WB	unk	Calcium influx	TRPC3 DISC1	Proliferation	[97]
	Cell	SH-SY5Y IMR-32	qRT-PCR WB	unk	Calcium influx	SK3	Proliferation migration	[98]

*unk*  unknown; *WB*  Western Blot

#### The role and mechanism of syncytin-1 in neuroblastoma

**Fig. 3:**
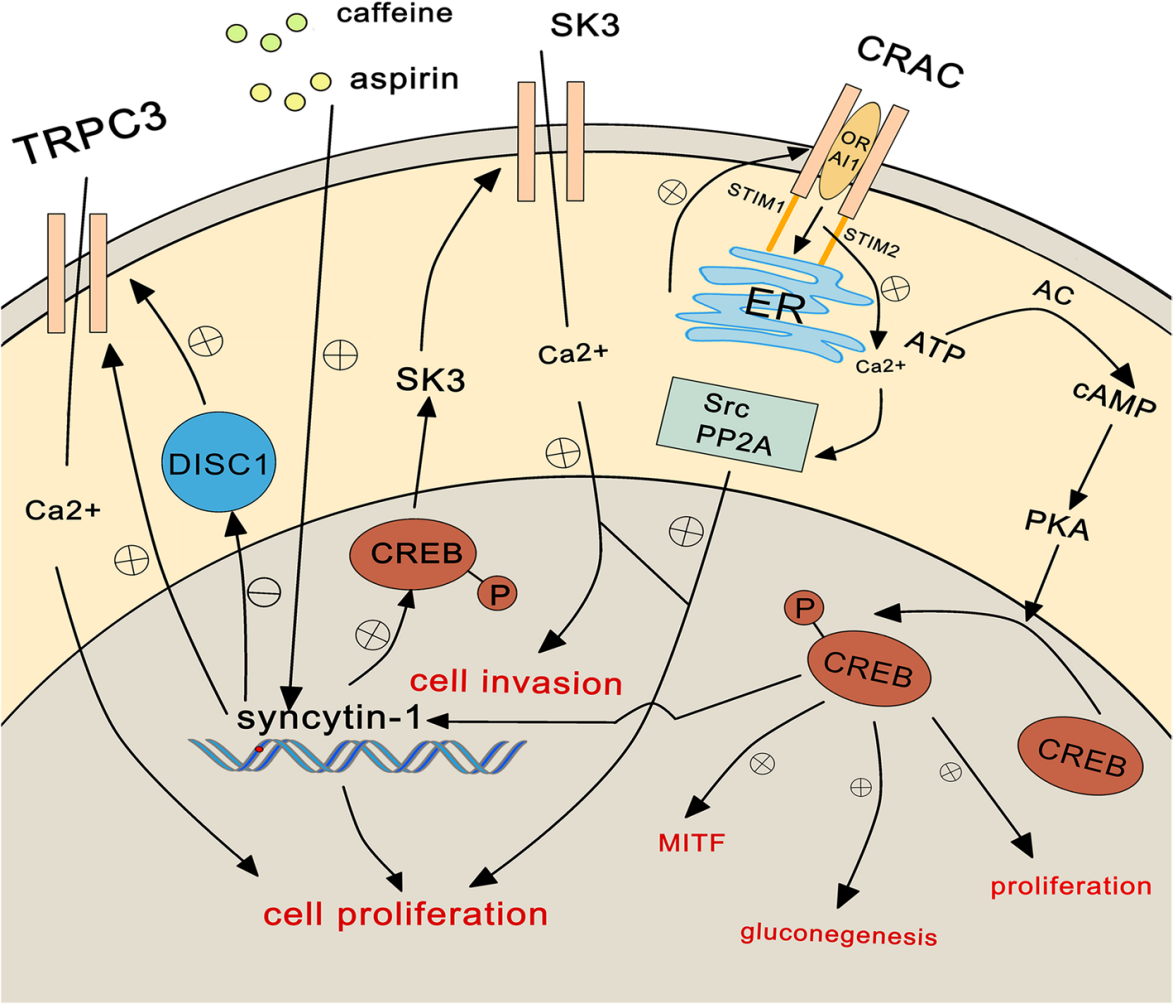
Molecular mechanisms and signal pathways of syncytin-1 overexpression in neuroblastoma (NB) Aspirin and caffeine can promote the transcription of syncytin-1, thereby promoting the proliferation of NB cells. The stromal interaction molecule 1 (STIM1) protein in the endoplasmic reticulum (ER) recognizes the depletion of stored calcium and activates the plasma membrane calcium ion channel (ORAI1) in the plasma membrane, establishing a calcium release-activated calcium channel (CRAC), causing calcium influx. The calcium that re-enters the cytoplasm is stored in the endoplasmic reticulum (ER). As the concentration of calcium in the cytoplasm increases, protein kinases such as Src and PP2A are activated, thus promoting cell proliferation. Syncytin-1 can induce calcium influx by activating the TRPC3 channel, SK3 channel and down-regulating DISC1 expression to promote the proliferation and invasion of NB cells. Phosphorylated CREB can also induce tumor cell proliferation, gluconeogenesis and promote microphthalmia-associated transcription factor (MITF) expression

Syncytin-1 induces tumorigenesis by multiple factors (Fig. 3). Firstly, the study found that aspirin and caffeine could increase the mRNA level and protein expression of syncytin-1 in the SH-SY5Y cell line. And the luciferase activity assay showed that caffeine could also induce the activation of HERV-W environmental promoter, improve the transcription level of syncytin-1, thus promoting the proliferation of NB cells [100]. Several studies suggested that caffeine could play roles in different pathways, such as releasing calcium stored [101], activating the phosphatidylinositol 3-kinase (PI3K)/protein kinase B (Akt) pathway [102], inhibiting cellular mTOR/P70S6K/4E-BP1 [103] or inducing vascular endothelial growth factor expression [104]. Secondly, treatment of the SK-N-DZ cell line with hypoxia-reoxygenation or DNA methylation inhibitor 5-azacytidine could increase the expression of syncytin-1 [99]. Thirdly, changes in the medium micro-environment can also affect the expression of syncytin-1. The expression of syncytin-1 in the NB cell line SH-SY5Y was significantly increased in serum-free stem cell medium, and RNA analysis found that the overexpression of HERV was associated with the overexpression of the immune checkpoint molecule CD200 in NB tumors [96], indicating that serum-free stem cell medium promotes NB cell invasion [105]. Therefore, caffeine, aspirin, oxygen tension, DNA methylation and the medium micro-environment changes both can affect the expression of syncytin-1.

Syncytin-1 affects NB progression through multiple molecular mechanisms. Store-operated calcium entry mechanism is one of the mechanisms of calcium influx. The stromal interaction molecule 1 protein in the endoplasmic reticulum recognizes the depletion of stored calcium and activates the calcium release-activated calcium channel protein 1 in the plasma membrane, establishing a calcium release-activated calcium channel, causing calcium influx. Calcium re-entering the cytoplasm is stored in the endoplasmic reticulum. As the concentration of calcium in the cytoplasm increases, protein kinases such as tyrosine-protein kinase (Src) and protein phosphatase 2 (PP2A) are activated, thus promoting cell proliferation and other processes [106, 107]. Syncytin-1 can promote the proliferation of NB cells by affecting calcium influx. Whole-cell patch clamp experiments showed that in NB cell lines SH-SY5Y and IMR-32, syncytin-1 could promote calcium influx by directly activating transient receptor potential channel 3 (TRPC3) channels and indirectly activating TRPC3 channels by downregulating the expression of DISC1, thereby promoting cell proliferation [97]. And syncytin-1 could also activate the phosphorylated cyclic-AMP response binding protein (CREB) site on the small conductance Ca2+ -activated K+ channel protein 3 (SK3) promoter, causing downstream gene SK3 transcription, and inducing calcium influx via a voltage-independent pathway, finally causing proliferation and migration of NB cells [98]. CREB is phosphorylated and activated by PKA, inducing several physiological processes through the formation of homodimers or heterodimers, such as upregulating microphthalmia-associated transcription factor [108, 109], promoting gluconeogenesis [110] and the proliferation of cancer cells. Furthermore, different types of transient receptor potential channel proteins overexpression were also found in pancreatic, ovarian and lung cancers, and overexpression of voltage-gated calcium channels has been found in testicular, prostate, colorectal, gastric cancers [111]. All in all, syncytin-1 can cause calcium influx by acting on TRPC3, CREB, SK3 and so on, prompting the proliferation and migration of NB cells.

In summary, syncytin-1 overexpression can be induced by extracellular factors such as drugs and culture micro-environment, and syncytin-1 overexpression can also activate channels that promote calcium influx and thus have an effect on NB. These results illustrate that syncytin-1 has the potential to become a prognostic marker for NB, but more definite mechanisms need to be further investigated.

### The relation between syncytin-1 and endometrial cancer/endometriosis

Endometrial cancer (EnCa) is one of the most common malignant invasive cancers in the world, often occurring in women with postmenopausal bleeding [112]. Studies have shown that the expression of syncytin-1 in EnCa tissues and cells is higher than that in normal tissues and cells (Table 3). Strick et al. found that both steroid hormones and cAMP can induce a significant increase in syncytin-1 mRNA and protein expression in EnCa cells. Steroid hormone-induced syncytin-1 promotes cell proliferation, and syncytin-1 promotes cell–cell fusion in the absence of transforming growth factor β. Similarly, cAMP also induces syncytin-1 to promote cell–cell fusion [113]. In addition, hypomethylation of the HERV-W 5′LTR region can increase syncytin-1 expression, and the tumor staging and histological grading of EnCa can also have an impact on syncytin-1 expression [114]. The overexpression of syncytin-1 promotes EnCa cell proliferation by rapidly transitioning from G2 to the M phase. And it also promotes cell migration and invasion by inducing the expression of epithelial-mesenchymal transition (EMT)-related gene proteins, such as waveform protein and E-cadherin protein. This result suggested that syncytin-1 as a fusogenic gene could promote cell migration and invasion by inducing EMT progress. Kaplan–Meier analysis found that high syncytin-1 expression was often associated with poor survival and prognosis [115]. This suggests that syncytin-1 is involved in the early oncogenic process of EnCa.

**Table 3:** Relationship between high expression of syncytin-1 and endometrial cancer

Type of cancer	Specimen	Number of cases	Methods	Upstream factors	Downstream factors	Oncogenic effect	Relationship between high expression of syncytin-1 and endometrial cancer:		Ref.
							Clinical parameter	Relevance	
Endometrial cancer	Tissue	48/(E:24,N:24)	qPCR RT-PCR Northern Blot	Steroids CAMP	unk	Fusion proliferation	unk	unk	[113]
	Tissue	67/(E:38,N:29)	qPCR	Hypomethylation	unk	Proliferation	TNM staging	+	[114]
							Histological grading	+	
	Tissue cell	167/(E:130,N:37)	RT-PCR	unk	Waveform E-adhesion	Fusion proliferation migration	unk	unk	[115]

*unk* unknown; *E* endometrial cancer; *N* normal; *+* positive correlation

Endometriosis is an estrogen-dependent chronic gynecological disease, it is characterized by the presence of endometrial tissue outside its normal location [116]. Similar to EnCa, syncytin-1 may also influence the development of endometriosis lesions. Zhou et al. found that syncytin-1 is overexpressed in endometriosis tissues via hypomethylation in the LTR promoter region. The alternations in DNMT3B isoforms can result in hypomethylation of the syncytin-1 promoter, and the overexpression of syncytin-1 can upregulate the human somatomammotropin (hCS) gene [117]. In addition, the RNA levels of syncytin-1 were increased only in eutopic endometrium from patients with endometriosis [118]. This suggests that syncytin-1 may play an essential role in the morphological development of the human endometrium.

### Correlation between syncytin-1 and testicular cancer/seminoma

Testicular cancer is a common solid malignant tumor in young men. Testicular germ cell tumors (TGCT) account for about 98% of all testicular malignancies, and seminomas account for about 60% of TGCT [119]. Gimenez et al. found in the custom HERV genechip microarray that syncytin-1 mRNA expression was not observed in normal testicular tissues, while the expression of syncytin-1 was up-regulated in testicular cancer tissues [120]. Another study found that the mRNA levels of syncytin-1 in seminoma tissues were higher than those in seminoma-matched controls and non-seminoma GCTs. The expression of tet methylcytosine dioxygenase 1 is highly increased in most seminomas, which results in hypomethylation of the syncytin-1 promoter, increasing the expression of syncytin-1. The interaction of syncytin-1 and its receptor SLC1A4/SLC1A5, and the transcriptional factor glial cells missing transcription factor 1 (GCM1) are all able to influence most tumorigenesis, nevertheless, the interaction has not been found in TGCT or seminomas [121]. So, the overexpression of syncytin-1 may be associated with the development of testicular cancer and seminoma, but further research is needed.

### Syncytin-1 and other tumors

The expression of syncytin-1 is high in NB cell lines, EnCa, testicular cancer and seminoma, and also abnormally expressed in other tumors. Studies have shown that when cell apoptosis or necrosis, chromosomal DNA is cleaved into large amounts of circulating free DNA, which is released into the serum and plasma [122, 123]. In non-small cell lung cancer (NSCLC), the syncytin-1 gene is increased expression due to chromosome activation and nucleosome depolymerization. Thus, the circulating free DNA of syncytin-1 is increased in the serum of NSCLC patients [124]. And the expression of syncytin-1 was significantly higher in NSCLC tissues than in para-carcinoma tissues. The hypomethylation of the syncytin-1 promoter could promote the expression of syncytin-1 in NSCLC [32]. The transcription factor SP1 can promote the expression of syncytin-1 in NSCLC cells. And the downregulation of the SP1/Syncytin-1 axis can reverse the epithelial-mesenchymal transition process by inhibiting the activity of Akt and Erk1/2 signal pathways in NSCLC cells, inhibiting cell proliferation and migration, promoting cell apoptosis [125]. Our previous study found that the expression of syncytin-1 in urothelial cell carcinoma tissues of the bladder is more than in tumor-adjacent tissues. The transcriptional activator c-Myb interacts with the mutated 3'-LTR of syncytin-1, upregulating the levels of mRNA and protein of syncytin-1, inducing urothelial cell carcinoma tumorigenesis and tumor cell proliferation [126]. Syncytin-1 expresses in breast cancer cells, and the corresponding D-type retroviral receptor SLC1A5 expresses in endothelial cells. Syncytin-1 can facilitate breast cancer-endothelial cell fusions. The downregulation of syncytin-1 expression highly inhibits cell fusion. And inhibitory syncytin peptides can also inhibit cell fusion. Therefore, syncytin-1 could be a potential therapeutic target to inhibit tumor cell fusion [127]. The expression of syncytin-1 is higher in colorectal cancer tissues than in normal tissues [128]. Li et al. found that arsenic trioxide (ATO) can increase the expression of transcription factor GCM1, which in turn increases the expression of syncytin-1 and its receptor SLC1A5, mediating cell fusion, inducing the formation of the polyploid giant cancer cells (PGCCs) in colorectal cancer, promoting the migration, proliferation and invasion of colorectal cancer cells. The expression level of syncytin-1 gradually increased with tumor grades increasingly. It was thought that the expression of syncytin-1 could be closely related to the pathological grade, clinical stage and distant metastasis of colorectal cancer tissues [129]. Syncytin-1 is also expressed in mycosis fungoides, a primary cutaneous T-cell lymphoma [130, 131]. Interestingly, the mRNA and protein levels of syncytin-1 in pancreatic cancer are lower than in normal tissues. And the decrease of syncytin-1 expression is related to the hypermethylation of CpG sites on 5′LTR [132]. Taken together, the expression of syncytin-1 is high in NSCLC, urothelial cell carcinoma tissues of the bladder, breast cancer and colorectal cancer, but low in pancreatic cancer. And syncytin-1 has the potential to be a molecular marker for their diagnosis in the future.

## Syncytin-2 and diseases

Syncytin-2 is an env glycoprotein of the HERV-FRD family. It plays an important role in placental development through its involvement in the formation of the syncytiotrophoblast [133]. The abnormal expression of syncytin-2 has also been associated with human diseases. The MFSD2A is a syncytin-2-mediated cell fusion cognate receptor. Studies have found that syncytin-2 is expressed in the breast cancer cell line MCF-7. Interestingly, the ectopic expression of the transcription factor GCM1 can upregulate the activity of the MFSD2A promoter, promote the expression of MFSD2A genes, and also bind to GCM1-binding sites (GBSs) in syncytin-2 gene (SYN2GBS) at the CpG site of 5'-LTR to induce syncytin-2 hypomethylation, transactivate the promoter of syncytin-2, and promote its transcription. The interaction of syncytin-2 and MFSD2A can eventually promote the fusion of MCF-7 cells [134]. Furthermore, the high expression of syncytn-2 is also associated with colorectal cancer [128], EnCa [114] and seminoma [121]. Therefore, the physiological and pathological role of syncytin-2 predicts that it could become an important molecular biological marker in the future.

## Conclusion

Syncytin-1 can promote the fusion of villous cytotrophoblast into syncytiotrophoblast, and play an essential role in human placenta development. And it also promotes tumor cell proliferation, anti-apoptotic, invasion, and migration through its fusogenic and non-fusogenic activities. Syncytin-1 abnormally expressed in placental development related diseases and tumors, including PE, infertility, IUGR, hydatidiform moles, NB, EnCa, endometriosis, testicular cancer, seminoma and NSCLC. Caffeine, aspirin, medium micro-environment, steroid hormones, cAMP, PPARγ/RXRα agonists and inhibitors, ATO, DNA methylation levels can all affect the transcription level of syncytin-1. And syncytin-1 can also affect various signal pathways to induce tumorigenesis, such as TRPC3, DISC1, SK3, Akt and Erk1/2, promoting the proliferation and invasion of tumor cells and improving the ability of distant metastasis. The following points need to be further considered deeply in this review. (1) Syncytin-1 is highly expressed in both NB, EnCa, endometriosis, testicular cancer, seminoma, NSCLC, urothelial cell carcinoma tissues of the bladder and colorectal cancer. But it is low expressed in pancreatic cancer, infertility and IUGR. Though studies have shown that syncytin-1 expression is associated with PE, high or low expression of syncytin-1 in PE remains controversial. So, it is necessary to further explore the more specific molecular mechanisms of syncytin-1 through experiments. (2) The expression of syncytin-1 is high in NB, EnCa, testicular cancer and other diseases, can syncytin-1 be directly used as a diagnostic marker for diseases? (3) Syncytin-1 is a membrane glycoprotein, which may inhibit tumorigenesis by applying inhibitor drugs to inhibit the activity and signal pathways of syncytin-1 and its receptors. In conclusion, whether syncytin-1 can become a new biological marker and potential therapeutic target and whether it can be more effective than the currently confirmed target requires further experimental and clinical research.

## Future direction

Molecularly targeted therapy has increasingly become a research hotspot. Although syncytin-1 plays a role in placental development related diseases and tumors, its molecular mechanisms and signal pathways need further study. Syncytin-1 is a membrane glycoprotein, so future studies could be focused on exploring antibody inhibitor drugs targeting syncytin-1 and its receptors, to inhibit the occurrence of diseases. Furthermore, studies have shown that syncytin-1 can be detected in the blood, which may serve as a diagnostic marker in the future. Syncytin-1 can participate in the formation of placental syncytiotrophoblasts through cell fusion, and could also play an important role in proliferation, migration and invasion of tumor, so its specific mechanisms need to be further explored in the future.

## Data Availability

Not applicable.
